# Drug-induced Stress Granule Formation Protects Sensory Hair Cells in Mouse Cochlear Explants During Ototoxicity

**DOI:** 10.1038/s41598-019-48393-w

**Published:** 2019-08-29

**Authors:** Ana Cláudia Gonçalves, Emily R. Towers, Naila Haq, John A. Porco, Jerry Pelletier, Sally J. Dawson, Jonathan E. Gale

**Affiliations:** 1 UCL Ear Institute, University College London, 332 Gray’s Inn Road, WC1X 8EE London, UK; 2 Department of Chemistry, Center for Molecular Discovery, Boston University, Boston, MA 02215 USA; 3 Department of Biochemistry, McGill University, Montreal, QC H3G 1Y6 Canada

**Keywords:** Mechanisms of disease, Hair cell

## Abstract

Stress granules regulate RNA translation during cellular stress, a mechanism that is generally presumed to be protective, since stress granule dysregulation caused by mutation or ageing is associated with neurodegenerative disease. Here, we investigate whether pharmacological manipulation of the stress granule pathway in the auditory organ, the cochlea, affects the survival of sensory hair cells during aminoglycoside ototoxicity, a common cause of acquired hearing loss. We show that hydroxamate (-)-9, a silvestrol analogue that inhibits eIF4A, induces stress granule formation in both an auditory cell line and *ex-vivo* cochlear cultures and that it prevents ototoxin-induced hair-cell death. In contrast, preventing stress granule formation using the small molecule inhibitor ISRIB increases hair-cell death. Furthermore, we provide the first evidence of stress granule formation in mammalian hair cells *in-vivo* triggered by aminoglycoside treatment. Our results demonstrate that pharmacological induction of stress granules enhances cell survival in native-tissue, in a clinically-relevant context. This establishes stress granules as a viable therapeutic target not only for hearing loss but also other neurodegenerative diseases.

## Introduction

Hearing loss is the most common sensory deficit observed with ageing^1^. Recently, age-related hearing loss (ARHL) was identified as a major risk factor for cognitive impairment and dementia^2,3^, highlighting the potential for common molecular pathological mechanisms. A primary cause of acquired hearing impairment is the permanent loss of hair cells, the mechanoreceptors for sound transduction in the inner ear. Research into the mechanisms underlying hair cell death implicates cellular stress as a critical component^4^. Cellular stress in the inner ear can be elicited either by intrinsic stimuli, such as the metabolic stress resulting from the demands of sound detection and amplification; or by extrinsic stimuli, such as ototoxic drugs (e.g. aminoglycoside antibiotics, cisplatin) or noise exposure^5–7^. Critically, since mammalian hair cells do not regenerate, their damage or death results in permanent hearing loss^8^. Our understanding of how hair-cells respond to cellular stress remains limited and as a result there are currently no efficacious drug therapies available to address hearing loss.

Aminoglycoside antibiotics, despite their known ototoxic and nephrotoxic side effects, are still some of the most commonly used antibiotics worldwide^9^. In the inner ear, aminoglycosides enter hair cells via the mechanotransduction channels^10,11^. Once inside the cells, aminoglycosides are thought to cause the formation of free radicals and subsequent activation of pro-apoptotic pathways and these are thought to be the primary cause of cell toxicity, although recent data indicates other possible mechanisms^9,12–14^. Previous work from our laboratory revealed that aminoglycoside exposure causes the assembly of stress granules in hair cells *in-vitro*^15^.

Stress granules (SGs) are membrane-free aggregates of mRNA and RNA-binding proteins that form during cellular stress. By controlling the fate of mRNAs, SGs play a key role in the post-transcriptional regulation of gene expression during stress^16^. Although this regulatory role is widely understood to be pro-survival there are examples where persistence of SGs has been associated with cell damage or death. Dysfunction of prion-like, low-complexity domains in certain RNA-binding proteins involved in SG-formation delay SG-disassembly, promoting the persistence of non-dynamic, insoluble SGs. These altered biophysical properties have been associated with the pathology of neurodegenerative diseases such as ALS, Alzheimer’s and Huntington’s diseases^17–19^.

Here, we developed a protocol for the pharmacological manipulation of SGs first in an inner ear-derived cell line and then in a mammalian intact-organ preparation, and investigated how modulating the SG-pathway affects hair-cell survival during ototoxicity. Hydroxamate (-)-9, a silvestrol-analogue that inhibits translation initiation through disruption of the protein translation eukaryotic initiation factor 4A (eIF4A)^20^, is used to trigger SG-formation and the small molecule Integrated Stress Response Inhibitor (ISRIB), that potentiates protein translation through stabilisation of the eukaryotic initiation factor 2B (eIF2B)^21^, is used to reduce SG-formation. We find that hydroxamate (-)-9 can significantly increase hair-cell survival during aminoglycoside-induced cochlear ototoxicity. Furthermore, we show that SGs form in the cochlea *in-vivo* in response to systemic application of aminoglycosides. Our results demonstrate, for the first time in native tissue, that induction of SGs during cellular stress is a pro-survival mechanism and that SGs, and regulators of SGs, are emerging as excellent targets for therapeutic intervention during ageing and neurodegeneration.

## Results and Discussion

### Pharmacological manipulation of SGs in UB/OC-2 cells and mouse cochlear explants

To date, much of our knowledge about SG-dynamics arises from studies in cell lines, and thus the properties of SGs in native tissue are still poorly understood. Our previous research investigating Pou4f3-regulation of the RNA binding protein Caprin-1, indicated that SGs could play an important role in the maintenance and survival of hair cells. Given the recent identification of a potential role for SGs in neurodegeneration^17–19^, we sought to explore the role of SGs in hair-cell survival during stress, namely whether pharmacological manipulation of SG-formation can protect against cell damage during ototoxicity. In this context, the auditory system is a model tissue in which to explore the role of SGs in cell survival during whole organ-level stress and neurodegeneration.

**Figure 1:**
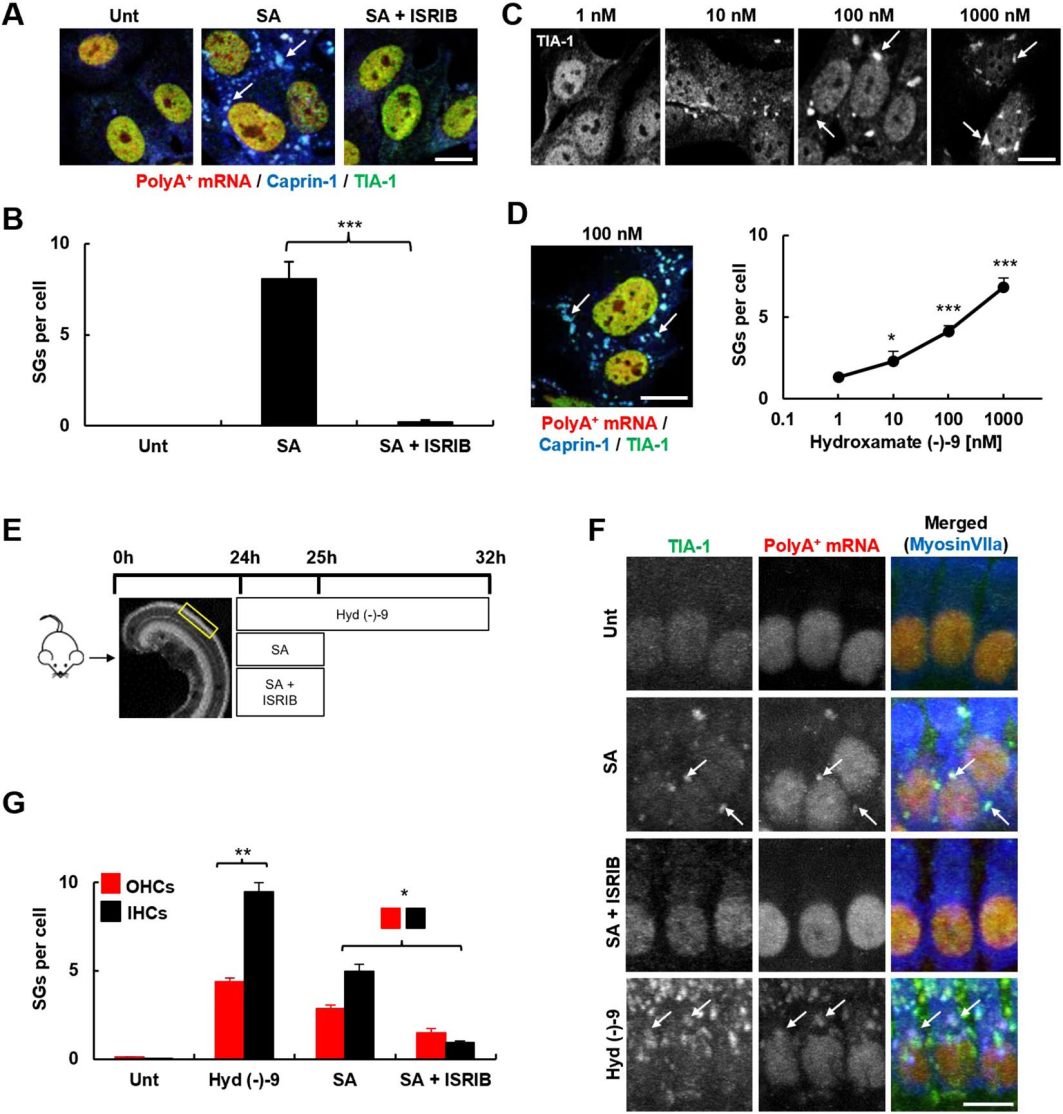
Pharmacological manipulation of SGs in UB/OC-2 cells and mouse cochlear explants. **(A)** UB/OC-2 cells treated with 0.5 mM sodium arsenite for 1 h in the absence or presence of 200 nM ISRIB. RNA-immuno-FISH detects polyA^+^ mRNA and Caprin-1 and TIA-1 proteins. Arrows, SGs in cells. **(B)** Quantification of the number of arsenite-induced SGs in UB/OC-2 cells in the presence or absence of ISRIB. **(C)** UB/OC-2 cells treated with increasing concentrations of hydroxamate (-)-9 for 8 h. Arrows, TIA-1-positive SGs. RNA-immuno-FISH in D (left panel) shows triple (polyA^+^ mRNA, Caprin-1 and TIA-1)-positive SGs with 100 nM hydroxamate (-)-9, scale bar = 10 µm. (**D**, right panel) Dose-dependent effect of hydroxamate (-)-9 on SG-formation. **(E)** Experimental paradigm used in P3 mouse cochlear explants to pharmacologically modulate SG-formation in native hair cells. No recovery was used in these studies, and cells were fixed after treatments. **(F)** Explants treated with 0.5 mM arsenite for 1 h in the absence or presence of 200 nM ISRIB or for 8 h with 100 nM of hydroxamate (-)-9 at 37 °C. PolyA^+^ mRNA, TIA-1 and MyosinVIIa were detected using RNA-immuno-FISH. IHCs are shown. Arrows, examples of SGs in hair cells. **(G)** Quantification of the number of SGs generated in hair cells following ISRIB and hydroxamate (-)-9 application. Basal coil cochlear explants were used and images were acquired from the mid-region of the coils. Error bars represent SEM (n = 9 for all conditions). *p < 0.05,**p < 0.01,***p < 0.001 for all conditions. Scale bars for all, 10 µm.

To assess SG-formation in the context of auditory cells and optimise drug treatment protocols, we first used UB/OC-2 cells, a cell line derived from the mouse inner ear^22^. We developed an RNA-immuno-FISH protocol to confirm polyA^+^ mRNA localization within SGs together with RNA-binding proteins TIA-1 and Caprin-1. In untreated UB/OC-2 cells, polyA^+^ mRNA and TIA-1 are located within the nucleus and cytoplasm and Caprin-1 is located predominantly in the cytoplasm (Fig. 1A). Following oxidative stress induced by arsenite exposure, we observe cytoplasmic aggregation of polyA^+^ mRNA, Caprin-1 and TIA-1, characteristic of SGs (Fig. 1A, arrows). Arsenite induces the formation of an average of 9 SGs/cell, significantly more than untreated cells (<1/cell, *p* < 0.001, Fig. 1B), and SGs range from 1.5 to 3.0 µm^2^ in size. This is consistent with the number and size of the SGs reported previously by others in HeLa, HEK293 and U2OS cells^23–25^, and shows that the SGs generated in UB/OC-2 cells are comparable to those described in other cell lines.

Using this arsenite paradigm we next investigated whether SG-formation could be pharmacologically modulated using either ISRIB or hydroxamate (-)-9. Promoting eIF2B activity using ISRIB almost completely prevents the formation of SGs during arsenite stress, such that the cellular distribution of polyA^+^ mRNA, Caprin-1 and TIA-1 is almost identical to that observed in unstressed cells (Fig. 1A). SG-formation is reduced by 97% when arsenite is administered in the presence of ISRIB (Fig. 1B, *p* < 0.001). In contrast, exposure to hydroxamate (-)-9 for 8 h results in a dose-dependent increase in the number of SGs per cell (Fig. 1C). When used at 100 nM, hydroxamate (-)-9 treatment results in similar numbers of polyA^+^ mRNA/TIA-1/Caprin-1-positive SGs per cell as induced by arsenite, with an average size of 1.3 µm^2^ (Fig. 1C,D).

To investigate the dynamics of SG-formation in native hair cells, we used cochlear explants from postnatal day 3 mice and applied the same stress paradigms and drug protocols developed in UB/OC-2 cells (Fig. 1E). Cochlear explants are unique multicellular organ preparations that allow the investigation of complex cell-to-cell signalling pathways and the development of toxicity assays in an intact mammalian tissue. In the organotypic cochlear explants there are two types of highly specialised receptor cells, the inner (IHCs) and outer hair cells (OHCs).

Arsenite induces the formation of polyA^+^ mRNA and TIA-1-positive SGs in both IHCs and OHCs (Fig. 1F, arrows). After 1 hour, an average of 5 and 3 SGs formed per IHC and OHC, respectively (Fig. 1G, *p* < 0.05), with an individual estimated volume of 0.4 µm^3^. When explants are treated with arsenite in the presence of ISRIB to stabilise eIF2B function and inhibit SG-formation during stress, we observe a significant reduction in the number of SGs formed in IHCs and OHCs, to 18% and 48% respectively, of the number formed with arsenite alone (Fig. 1F,G, *p* < 0.05). The disruption of eIF4A by exposure to hydroxamate (-)-9 induces SG-formation in the cytoplasm of hair cells, as shown by the aggregation of TIA-1 with polyA^+^ mRNA (Fig. 1F, arrows). On average, hydroxamate (-)-9 induces the formation of 9 and 4 SGs in IHCs and OHCs, respectively, averaging 0.5 µm^3^ in size (Fig. 1G, *p* < 0.001). Overall, the number and size of SGs formed in native tissue were similar to those observed in UB/OC-2 cells. To our knowledge, our study is the first to provide a detailed description of SGs found in mammalian tissue, in terms of number and size. Further work is required to explore the differences between regular dynamics of SG-assembly and dysregulation during neurodegeneration in native tissue. Importantly, during our experiments, the highly organised structure of the organ of Corti, with three rows of OHCs and a single row of IHCs, was maintained, suggesting that exposure to ISRIB or hydroxamate (-)-9 alone does not disrupt the sensory epithelium (Supplementary Fig. S1).

### Pharmacological induction of SGs promotes outer hair cell survival following aminoglycoside exposure

**Figure 2:**
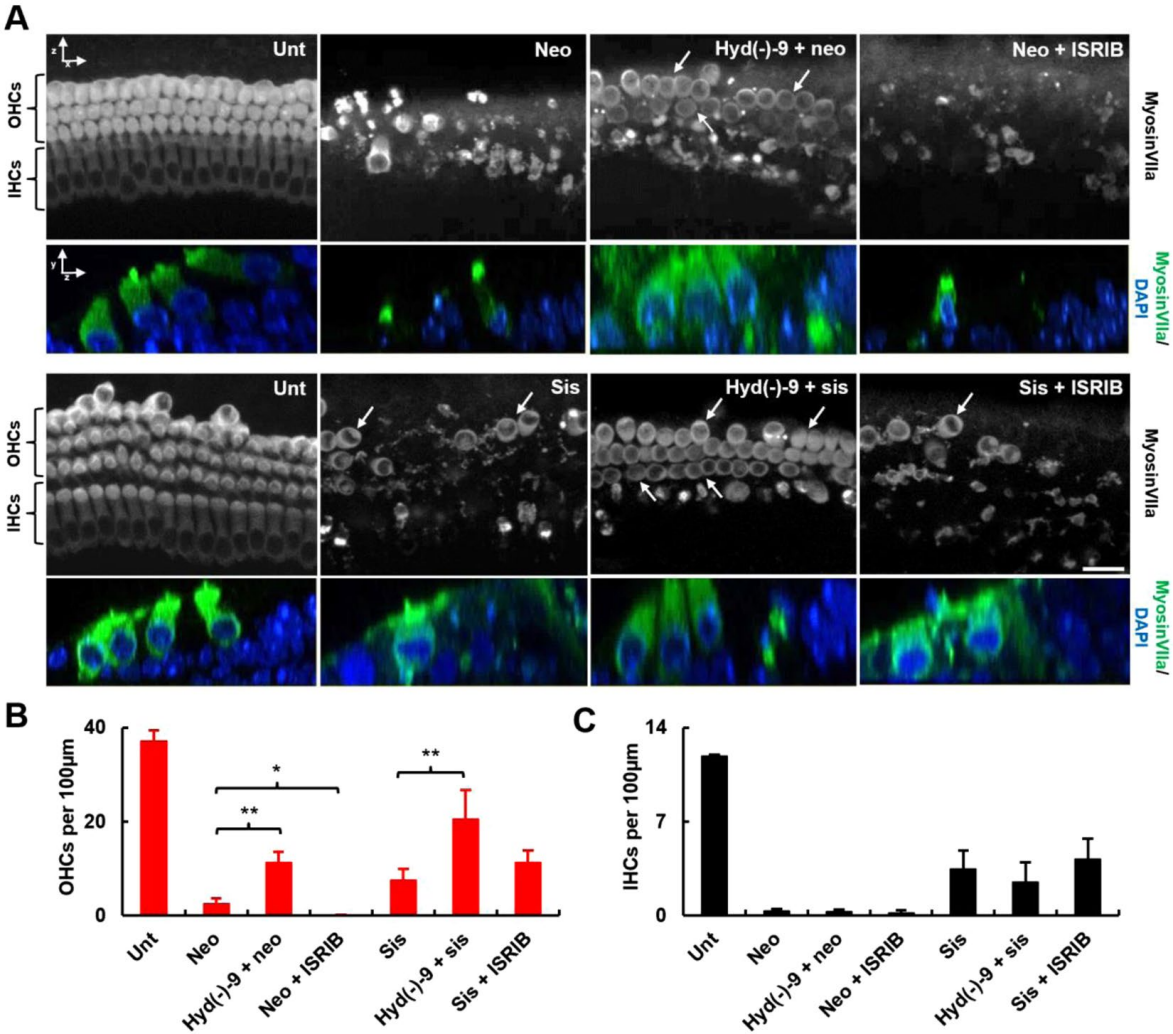
Hydroxamate (-)-9 increases outer hair cell survival in response to aminoglycoside toxicity. (**A)** Cochlear explants were subjected to either 1 mM neomycin for 6 h or 500 µM sisomicin for 1 h, and allowed to recover for 48 h. Hydroxamate (-)-9 was applied at 100 nM for 14 h prior to aminoglycoside exposure. ISRIB (200 nM) was co-applied with neomycin or sisomicin and then maintained in an aminoglycoside-free medium for 24 h. All samples were allowed to recover for 48 h in an aminoglycoside-free medium before fixation. Anti-myosinVIIa used to label IHCs and OHCs. Arrows, examples of surviving OHCs after aminoglycoside treatment. All treatments were performed using the mid-sections of the basal cochlear coils, where the strongest effects on hair cell loss after neomycin or sisomicin application are observed. Images are average intensity Z-projections from confocal stacks. Lower, colour merge panels are orthogonal (YZ) projections from the confocal stacks. Scale bar = 10 µm. **(B, C)** Graphs show average number of surviving OHC and IHC, respectively. Error bars, SEM. *p < 0.05 **p < 0.01, Student’s *t*-test. Data were obtained from at least three separate experiments. Each n represents a separate basal cochlear explant as follows: control (n = 6), neomycin (n = 7), sisomicin (n = 7) hydroxamate (-)-9 + neomycin (n = 9), hydroxamate (-)-9 + sisomicin (n = 7), neomycin + ISRIB (n = 4), sisomicin + ISRIB (n = 9).

To investigate whether manipulating SG-formation can promote hair-cell survival during toxic cellular stress, we used hydroxamate (-)-9 and ISRIB to modulate eIF4A and eIF2B activities during aminoglycoside toxicity. Neomycin, an aminoglycoside antibiotic that causes hair-cell death principally by apoptosis^26^, was applied for 6 h at 1 mM. Hair-cell survival was determined using myosinVIIa-labelling (Fig. 2A); 48 h after the end of the neomycin exposure, only 2.5% of IHCs and 6.5% of OHCs remain (Fig. 2B,C, *p* < 0.001). Treatment with hydroxamate (-)-9 to trigger SG-formation before neomycin exposure improves OHC survival by > 4-fold to 29% (Fig. 2A,B, *p* < 0.01). Conversely, when ISRIB is incubated with neomycin, almost no intact OHCs are present after 48 h (Fig. 2A,B, *p* < 0.001 versus neomycin-alone). The effects of either hydroxamate (-)-9 or ISRIB treatment on IHC survival were not significant (Fig. 2A,C).

Since hydroxamate (-)-9 pre-treatment protects OHCs from exposure to neomycin, we tested if these results were replicated with another aminoglycoside antibiotic, sisomicin, an aminoglycoside that has been used in other studies due to its potency and consistency^27,28^. A one-hour exposure to 500 µM sisomicin is enough to cause a significant hair-cell loss resulting in 27% of IHCs and 19% of OHCs surviving 48 h later (Fig. 2A–C, *p* < 0.001 for both IHCs and OHCs, versus untreated). Again, pre-treatment with hydroxamate (-)-9 protects OHCs from sisomicin-induced death with ~3-fold increase in OHC survival compared to sisomicin-alone (Fig. 2A,B, *p* < 0.01). Inhibiting SG-formation with ISRIB did not significantly affect the number of surviving IHCs (36%) or OHCs (29%) compared to sisomicin-alone (Fig. 2A–C).

**Figure 3:**
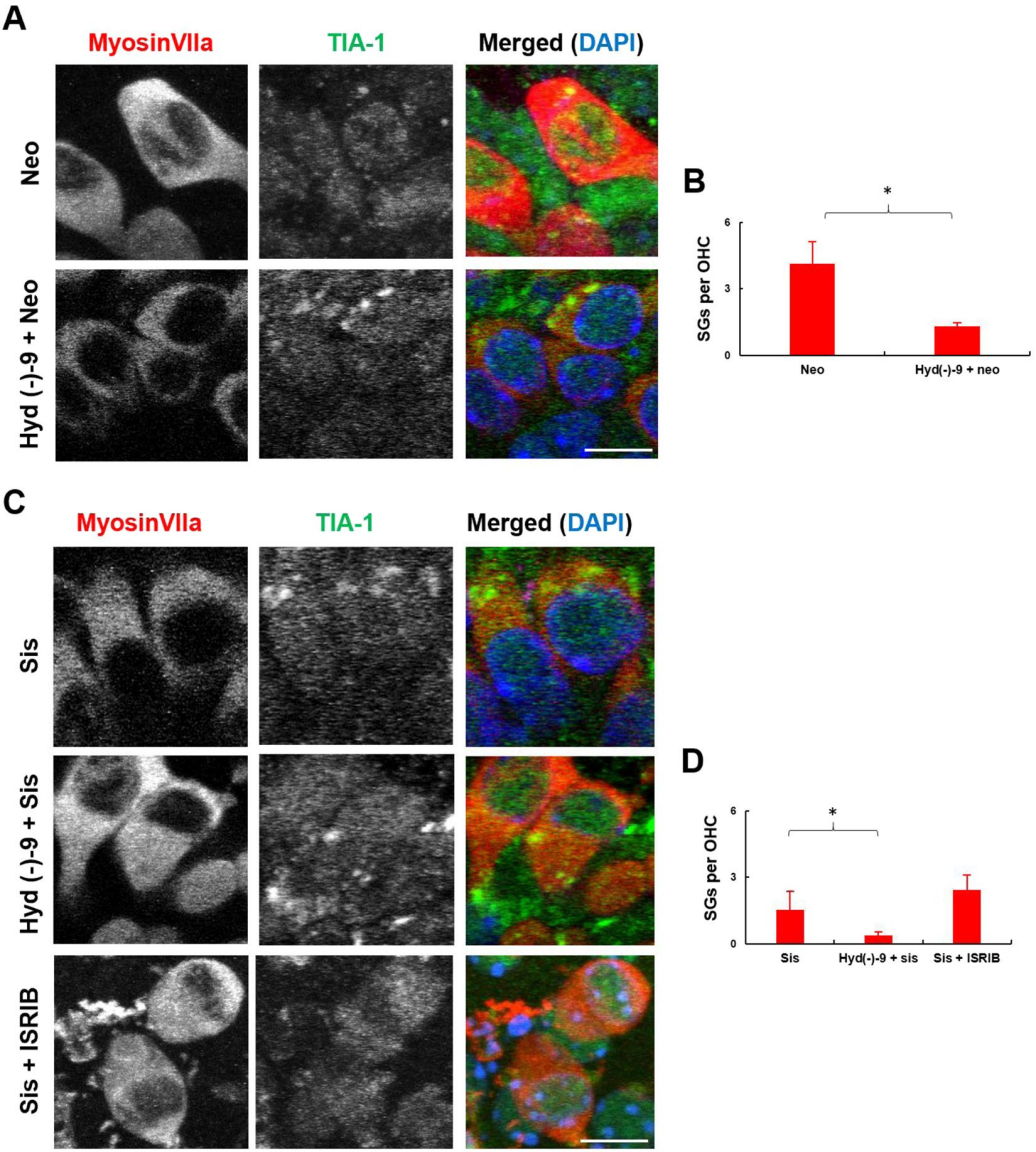
Surviving OHCs contain SGs after aminoglycoside treatment. Cochlear explants were subjected to either 1 mM neomycin for 6 h (**A**) or 500 μM sisomicin for 1 h (**C**) and allowed to recover for 48 h. Hydroxamate (-)-9 was applied at 100 nM for 14 h prior to aminoglycoside exposure. ISRIB (200 nM) was co-applied with neomycin or sisomicin and kept in an aminoglycoside-free medium for 24 h. All samples were allowed to recover for 48 h in an aminoglycoside-free medium before fixation. Images show examples of surviving OHCs containing SGs at the end of the experiment. HCs were labelled with anti-myosin VIIa and SGs with anti-TIA-1. Basal coil cochlear explants were used and images (134 × 104 μm) were acquired from the mid-region of the coils. Images shown are 25 × 25 μm regions to show subcellular detail. Bar graphs show quantification of SGs in surviving OHCs following neomycin (**B**) and sisomicin (**D**) treatments. Note, SGs were quantified in cells across the whole 134 × 104 μm image. Error bars represent SEM. Each n represents a separate cochlear explant as follows: control (n = 6), neomycin (n = 7), sisomicin (n = 7) hydroxamate (-)-9 + neomycin (n = 9), hydroxamate (-)-9 + sisomicin (n = 7), neomycin + ISRIB (n = 4), sisomicin + ISRIB (n = 9). *p < 0.05. Scale bar for all, 10 μm.

We quantified the number of SGs in the surviving OHCs at the end of the 48 hour recovery period since we observed a protective effect on those cells. After neomycin exposure, surviving OHCs contain an average of 4.1 ± 1.0 SGs per cell compared to 1.3 ± 0.2 SGs per cell when they were pre-incubated with hydroxamate (-)-9 (Fig. 3A,B, *p* < 0.05). Surviving OHCs that have been exposed to hydroxamate (-)-9 prior to sisomicin also contain less SGs than those exposed to sisomicin alone (an average of 0.3 ± 0.2 compared with 1.5 ± 0.8 SGs per cell, respectively, Fig. 3C,D). One possibility is that hydroxamate (-)-9-mediated SG-formation alters the translational profile, i.e. mRNA profile, of hair cells such that their response to ototoxic insult is optimised for survival and they can recover faster after the end of the insult. Consistent with this we observe a reduced number of SGs per cell in the surviving hair cells and hair cells from explants that were not treated with hydroxamate (-)-9 contain more SGs per cell. The persistence of SGs after a stress insult is likely to be important for the long term survival of the cell and is worthy of further exploration.

Since aminoglycosides are known to enter hair cells via the mechanotransducer channels^10,11^, we wanted to confirm that hydroxamate (-)-9 does not prevent aminoglycoside entry. FM1-43 is a divalent fluorescent vital dye that mimics the entry of aminoglycosides by entering hair cells via the mechanotransducer channel and can be used to test whether the channel is still functional^10^. Hair cells are still functional and can be loaded with FM1-43 after hydroxamate (-)-9 exposure (Supplementary Fig. S2), indicating that hydroxamate (-)-9 is not toxic, and moreover that its protective effects are not due to blockade of the mechanotransducer channels. Altogether, our results show an increase in OHC survival in cochlear explants treated with hydroxamate (-)-9 before exposure to two different aminoglycosides (Fig. 2). Since hydroxamate (-)-9 disrupts eIF4A at the translation initiation step^20^, this triggers SG-formation, through translation inhibition. By targeting eIF4A, hydroxamate (-)-9 acts well downstream the stress-cascade pathway, thus minimising side effects on other cellular pathways. Hence, this suggests that the protective effect of hydroxamate (-)-9 on hair cells results from the eIF4A-mediated enhancement of SG-formation. In contrast, inhibiting SG-formation using ISRIB causes a further reduction in hair-cell survival following neomycin exposure. These data show, for the first time, that SGs play a role in promoting hair-cell survival during stress in the mammalian auditory system. In a wider context, our results are some of the first direct evidence from an intact mammalian tissue that the formation of SGs during cellular stress is, as might be expected, a protective response. Hence, pharmacological intervention to promote SG-formation may have therapeutic benefits in patients undergoing aminoglycoside-treatment and potentially those affected by ARHL or neurodegeneration. Aminoglycosides primarily target mitochondrial and cytoplasmic ribosomal proteins^29^, triggering translational arrest, thereby activating the JNK-apoptotic pathway^12^. The protective effect of triggering SG-formation prior to aminoglycoside exposure is consistent with a ribotoxic-stress mechanism, possibly involving SG-mediated sequestration of pro-apoptotic factors^30^ and ribosomal proteins^31^. Simultaneously, SG-mediated translation of stress-responsive genes could prime the OHCs for aminoglycoside-induced cellular stress.

### *In-vivo* aminoglycoside administration triggers SG-formation in the cochlea

We next investigated whether the SG-pathway is activated in the cochlea following aminoglycoside administration *in-vivo*. We applied a well characterised treatment regimen^32^ injecting a single dose of the aminoglycoside kanamycin followed by the loop diuretic, bumetanide. Ototoxicity, noise or ageing all affect the cochlea in a base-to-apex gradient, with cells in the basal, high-frequency coil of the cochlea, being primarily affected with a subsequent progression of cell death towards the lower-frequency apical coil. This manifests as a high-to-low frequency gradient of sensitivity in auditory pathology, and in addition, typically, OHC loss precedes IHC loss in cochlear degeneration^33,34^.

**Figure 4:**
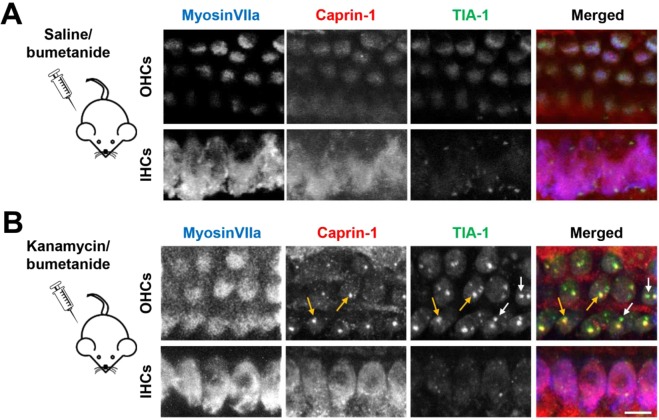
*In-vivo* aminoglycoside exposure generates SGs in hair cells. Post-natal day 18 mice were treated with a single intraperitoneal injection of either saline (control) at 1 mg/g body weight (**A**) or kanamycin at 1 mg/g body weight (**B**) followed by the loop diuretic bumetanide at 0.05 mg/g after 40 min. Cochleae were collected 24 h post-treatment. Yellow arrows in B indicate colocalisation of Caprin-1 and TIA-1 within SGs. White arrows in B indicate SGs positive for TIA-1 alone. MyosinVIIa labels IHCs and OHCs. All images were acquired from the apical cochlear coil where, after 24 hours, hair cells were still present using this drug protocol, so SG formation could be assessed. Images are maximum intensity projections of confocal sub-Z-stacks. Images are representative of data from at least three separate mice for each condition. Scale bar = 10 µm for all.

Twenty-four hours after systemic kanamycin/bumetanide treatment in C57BL/6 mice, we observe OHC loss in the basal turn of the cochlea (data not shown) consistent with findings from CBA mice^32^. However, examination of the apical turn reveals no hair cell loss or nuclear abnormalities, so we used this area to assess the SG-response. Caprin-1 and TIA-1-positive granular structures are present in OHCs (Fig. 4B, yellow arrows), consistent with the properties of SGs described in explant cultures, in terms of SG protein composition, number and size (Fig. 1F). Although the majority of SGs are double-labelled for both RNA-binding proteins, we do observe TIA-1-positive SGs that are negative for Caprin-1 (Fig. 4B) indicating subtypes of SGs *in-vivo*. We do not observe robust SG-formation in IHCs using this protocol, suggesting that OHCs are undergoing a greater cellular stress at this time point, consistent with previous similar experiments^32,35^.

Our data indicate that hydroxamate (-)-9-induced SG-formation provides a significant hair cell protection, but why are OHCs protected but not IHCs? OHCs are more vulnerable to aminoglycosides, noise and also to ageing and may be exposed to more frequent stress than IHCs. It has been proposed that repeated SG assembly/disassembly from chronic cellular stress, can lead to reduced SG-mediated capacity to deal with stress^36^, which hydroxamate (-)-9 treatment would overcome. IHCs and OHCs may have different capacities for SG formation and perhaps there is a critical number of SGs that need to be formed in order to provide a protective effect. It is worth noting however that, hydroxamate (-)-9 generated almost twice as many SGs in IHCs compared to OHCs (Fig. 1G). Thus, given that we observe protection of OHCs rather than IHCs the reverse could be possible, such that an excess of SGs or moreover more stable SGs could lead to a shutdown of protein synthesis, thus compromising the ability of cells to make pro-survival proteins. Further studies in both cell lines and native tissue are needed to clarify this. We note that some studies indicate that aminoglycosides cause hair-cell death through caspase-dependent pathways^32^, whilst others suggest hair-cells die through necrosis^35^. Since SGs can regulate apoptosis by sequestering RACK, MTK, TRAF and JNK^30,37^, a scenario consistent with our data is that aminoglycoside-induced death in OHCs is more “apoptotic” than in IHCs, which could invoke a more autophagic cell-death. The different mechanisms underlying hair cell death remain to be tested.

**Figure 5:**
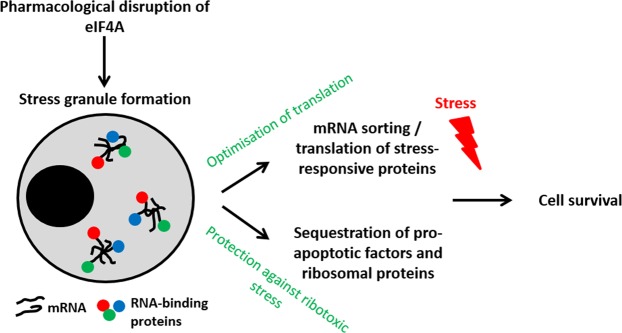
Model for the protective effects of SG-induction. Drug-induced disruption of eIF4A prior to stress exposure promotes SG-formation. SGs optimise the translation of stress-responsive RNAs, so that the cell’s response is primed when subsequently exposed to stress. At the same time, SGs sequester both pro-apoptotic factors^30^, inhibiting their translation, and also ribosomal subunits^31^ which protects against ribotoxic stress during ototoxicity. When the stress is overcome, ribosomal subunits that were part of SGs are functional and ready to re-initiate translation.

In summary, we show for the first time in intact mammalian tissue that induction of SG-formation through disruption of eIF4A using a novel silvestrol-analogue can promote cell survival during stress. We propose a model in which drug-induced disruption of eIF4A, and subsequent SG-formation promotes cell survival through: (1) promoting the translation of stress-responsive proteins, so the stress response is already primed towards cell survival; and/or (2) sequestration of pro-apoptotic factors and ribosomal sub-units, consistent with a ribotoxic-stress-based cell death mechanism (Fig. 5). Both our *in-vivo* and *ex-vivo* data suggest that the SG-pathway is activated by aminoglycoside exposure and can regulate the hair-cell response to stress. Our *ex-vivo* data in mouse cochlear explants suggest that SG-formation during cellular stress in mammalian cells is a pro-survival mechanism. Moreover, these data reveal an opportunity to protect sensory cells by pharmacological induction of SGs, pinpointing SGs as a potential therapeutic target. The findings are potentially relevant not only to cochlear stress and ARHL but also to other age-related neurodegenerative diseases.

## Materials and Methods

Mice were sacrificed according to Schedule 1 procedures as described in the United Kingdom (Scientific Procedures) Act of 1986, and approved by the UCL Biological Services Animal Ethics Committee. Cochlear explants from postnatal day 2–3 mice were cultured such as described previously^15^. Experiments were performed on explant cultures of basal cochlear coils only and images were acquired from the middle section of the basal coils. For arsenite treatment, mouse cochlear explants were incubated at 37 °C for 1 h with 0.5 mM sodium arsenite in DMEM F-12 medium supplemented with 1% FBS. ISRIB (Sigma) was used at 200 nM as described in ref.^21^.

UB/OC-2 cells, derived from the Immortomouse^TM^ (ref.^22^) were cultured at 33 °C under 5% CO2 atmosphere in Dulbecco’s modified Eagle’s medium (DMEM) supplemented with 10% fetal bovine serum (FBS) and 50 units/mL γIF, as described previously^15^. Hydroxamate (-)-9 was provided by Dr Jerry Pelletier (McGill University, Montreal) and Dr John Porco (Boston University, USA). In preliminary experiments on UB/OC-2 cells we tested a range of different incubation times (2, 4, 6 hours – data not shown) but found that a longer exposure of 8 hours was most effective. Hydroxamate (-)-9 was applied in DMEM F-12 medium supplemented with 1% FBS. Neomycin (Sigma) was applied for 6 hours at 1 mM and sisomicin (Sigma) was applied at 500 μM for 1 h. All samples were allowed to recover in an aminoglycoside-free medium for 48 h before fixation. All cochlear explant samples were fixed in 4% PFA in PBS for 30 minutes at room temperature and rinsed three times with PBS for 45 minutes. Samples were kept in PBS at 4 °C until further immunolabelling and analysis.

*In-vivo* aminoglycoside exposure was performed in 18 day old C57BL/6 mice, using the protocol described^30^. For the *in-vivo* experiments, images were acquired from the apical cochlear coil.

For RNA-immuno-FISH, decalcified inner ear tissue was permeabilised with −20 °C methanol for 10 min and rinsed twice with 2xSSC at 25 °C. Hybridisation was performed at 43 °C for 14 h in the dark in RNA hybridisation mixture containing 25%(v/v) formamide, 200 ng/μL salmon sperm DNA, 5xDenhardt’s solution, 50 mM sodium phosphate pH7, 1 mM EDTA, 2xSSC and 200 ng/μL of polydT-5’ probe (Eurofins). Primary-antibody detection was performed in blocking solution (0,5% Triton-X, 0.001% BSA, 1% serum) for 14 h at 4 °C and was against: TIA-1 (goat-ab; SantaCruz Biotechnology; 1:300), Caprin-1 (rabbit-ab; Proteintech Europe; 1:500) and myosinVIIa (rabbit-ab; Thermofisher; 1:500). Samples were incubated with the secondary-antibodies: donkey anti-(goat IgG) conjugated to Alexa Fluor-488 (SantaCruz Biotechnology); goat anti-(rabbit IgG) conjugated to Alexa Fluor-647 (Invitrogen), all 1:1000 concentration, in blocking solution for 2 h at 25 °C. Samples were imaged using either a Zeiss 510 NLO multi-photon upright confocal system using a 63x (1.0NA) immersion objective or on a Zeiss 510META inverted confocal system using a 63x (1.4NA) objective. A plugin for the Fiji/ImageJ image analysis software designated “SG counter” was used to quantify the number and size of SGs.

Statistical analyses were performed using unpaired, two-tailed Student’s *t* test or 1-way ANOVA with Tukey’s multiple comparisons correction (SPSS software).

### Study approval

All experiments using C57BL/6 mice were performed in accordance with the procedures as described in the United Kingdom (Scientific Procedures) Act of 1986. All experiments were approved and carried out under the Project Licence number PPL 70/8144.

## Supplementary information

supplementary information

## References

[1] Bowl Michael R., Dawson Sally J. (2018). Age-Related Hearing Loss. Cold Spring Harbor Perspectives in Medicine.

[2] Deal JA (2017). Hearing Impairment and Incident Dementia and Cognitive Decline in Older Adults: The Health ABC Study. J Gerontol A Biol Med Sci..

[3] Golub JS (2017). Observed Hearing Loss and Incident Dementia in a Multiethnic Cohort. J Am Geriatr Soc..

[4] Altschuler RA (2002). Stress pathways in the rat cochlea and potential for protection from acquired deafness. Audiology and Neuro-Otology..

[5] Lopez-Gonzalez MA (1999). Aminoglycosides activate oxygen metabolites production in the cochlea of mature and developing rats. Hear Res..

[6] Staecker H (2001). Oxidative stress in aging in the C57B16/J mouse cochlea. Acta Oto-Laryngol..

[7] Henderson D (2006). The role of oxidative stress in noise-induced hearing loss. Ear hearing..

[8] Edge AS, Chen ZY (2008). Hair cell regeneration. Curr Opi. Neurobiol..

[9] Forge A, Schacht J (2000). Aminoglycoside antibiotics. Audiology and Neurotology..

[10] Gale JE (2001). FM1-43 dye behaves as a permeant blocker of the hair-cell mechanotransducer channel. J Neurosci..

[11] Marcotti W (2005). The aminoglycoside antibiotic dihydrostreptomycin rapidly enters mouse outer hair cells through the mechano-electrical transducer channels. J Physiol..

[12] Francis SP (2013). A novel role of cytosolic protein synthesis inhibition in aminoglycoside ototoxicity. J Neurosci..

[13] Majumder P (2015). Cellular glutathione content in the organ of Corti and its role during ototoxicity. Front Cell Neurosci..

[14] O’Sullivan ME (2017). Towards the prevention of Aminoglycoside-related hearing loss. Front Cell Neurosci..

[15] Towers ER (2011). Caprin-1 is a target of the deafness gene Pou4f3 and is recruited to stress granules in cochlear hair cells in response to ototoxic damage. J Cell Sci..

[16] Anderson P, Kedersha N (2009). RNA granules: post-transcriptional and epigenetic modulators of gene expression. Mol Cell Biol..

[17] Chen Lihua, Liu Beidong (2017). Relationships between Stress Granules, Oxidative Stress, and Neurodegenerative Diseases. Oxidative Medicine and Cellular Longevity.

[18] Gopal PP (2017). Amyotrophic lateral sclerosis-linked mutations increase the viscosity of liquid-like TDP-43 RNP granules in neurons. PNAS..

[19] Mackenzie IR (2017). TIA1 Mutations in Amyotrophic Lateral Sclerosis and Frontotemporal Dementia Promote Phase Separation and Alter Stress Granule Dynamics. Neuron..

[20] Rodrigo CM (2012). Synthesis of rocaglamide hydroxamates and related compounds as eukaryotic translation inhibitors: Synthetic and biological studies. J Med Chem..

[21] Sidrauski C (2015). The small molecule ISRIB reverses the effects of eIF2 alpha phosphorylation on translation and stress granule assembly. eLife..

[22] Rivolta MN (1998). Auditory hair cell precursors immortalized from the mammalian inner ear. Proc R Soc Lond B..

[23] Souquere S (2009). Unravelling the ultrastructure of stress granules and associated P-bodies in human cells. J Cell Sci..

[24] Zurla C (2011). Characterizing mRNA interactions with RNA granules during translation initiation inhibition. PLoS ONE..

[25] McDonald KK (2011). TAR DNA-binding protein 43 (TDP-43) regulates stress granule dynamics via differential regulation of G3BP and TIA-1. Hum Mol Genet..

[26] Forge A, Li L (2000). Apoptotic death of hair cells in mammalian vestibular sensory epithelia. Hear Res..

[27] Huth ME (2015). Designer aminoglycosides prevent cochlear hair cell loss and hearing loss. Journal of Clinical Investigation..

[28] Scheibinger M (2018). Aminoglycoside Damage and Hair Cell Regeneration in the Chicken Utricle. JARO..

[29] Prokhorova I (2017). Aminoglycoside interactions and impacts on the eukaryotic ribosome. PNAS..

[30] Arimoto K (2008). Formation of stress granules inhibits apoptosis by suppressing stress-responsive MAPK pathways. Nat Cell Biol..

[31] Kedersha N (2002). Evidence that ternary complex (eIF2-GTP-tRNA(i)(Met))-deficient preinitiation complexes are core constituents of mammalian stress granules. Mol Biol Cell..

[32] Taylor RR (2008). Rapid hair cell loss: A mouse model for cochlear lesions. JARO..

[33] Schuknecht HF (1964). Further observations on the pathology of presbycusis. Arch Otolaryngol..

[34] Sha S-H (2001). Differential vulnerability of basal and apical hair cells is based on intrinsic susceptibility to free radicals. Hear Res..

[35] Jiang H (2006). Caspase-independent pathways of hair cell death induced by kanamycin *in vivo*. Cell Death Differ..

[36] Shelkovnikova TA (2017). Chronically stressed or stress-preconditioned neurons fail to maintain stress granule assembly. Cell Death Dis..

[37] Kim WJ (2005). Sequestration of TRAF2 into Stress Granules Interrupts Tumor Necrosis Factor Signaling under Stress Conditions Sequestration of TRAF2 into Stress Granules Interrupts Tumor Necrosis Factor Signaling under Stress Conditions. Mol Cell Biol..

