# Supplementary material for: Drug-induced Stress Granule Formation Protects Sensory Hair Cells in Mouse Cochlear Explants During Ototoxicity
Source: Sci Rep. 2019 Aug 29;9:12501. doi: 10.1038/s41598-019-48393-w (PMC6715625; doi:10.1038/s41598-019-48393-w)
Supplement: Supplementary file 1 — supplementary information [file 41598_2019_48393_MOESM1_ESM.pdf]

# Drug-induced Stress Granule Formation Protects Sensory Hair Cells in Mouse Cochlear Explants During Ototoxicity

Ana Claudia Goncalves[1], Emily R Towers[1], Naila Haq[1], John A Porco Jr[2], Jerry Pelletier [3], Sally J Dawson [1*] and Jonathan E Gale [1*]

1 = UCL Ear Institute, University College London, 332 Gray's Inn Road, WC1X 8EE, London UK.

2 = Department of Chemistry, Center for Molecular Discovery, Boston University, Boston, MA 02215.

3 = Department of Biochemistry, McGill University, Montreal, QC, H3G 0B1 Canada.

*= joint senior authors

**Corresponding authors**:

Jonathan Gale and Sally Dawson, UCL Ear Institute, 332 Gray's Inn Road, WC1X 8EE London, UK. j.e.gale@ucl.ac.uk and sally.dawson@ucl.ac.uk

**Supplementary information**

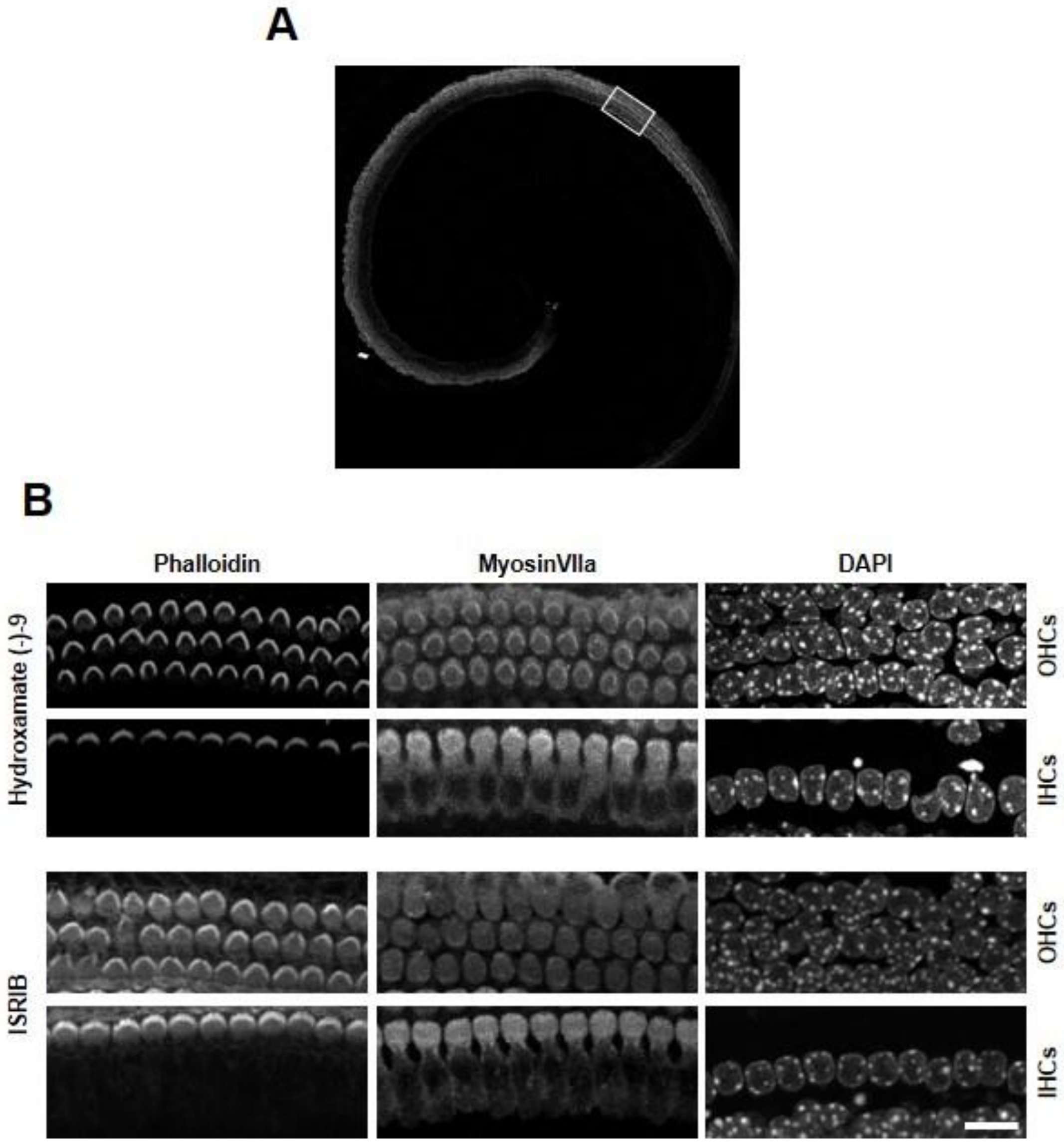


Supplementary figure 1 – **Neither ISRIB or hydroxamate (-)-9 affect the structure of the organ of Corti**. (**A**) Low magnification view of a mouse cochlear explant after dissection, showing the basal turn of the cochlea. Boxed area correspond to the mid of the basal turn, where images were collected for all the experiments performed in mouse cochlear explants. (**B**) Cochlear explants were subjected to either 100nM hydroxamate (-)-9 for 14h or 200nM ISRIB for 1h. Anti-myosinVIIa was used to label IHCs and OHCs, Phalloidin to label the F-actin filaments, and DAPI to assess chromatin structure. All images were acquired from mid-sections of basal cochlear coils. Images are average intensity Z-projections from confocal stacks. Scale bar=10µm.

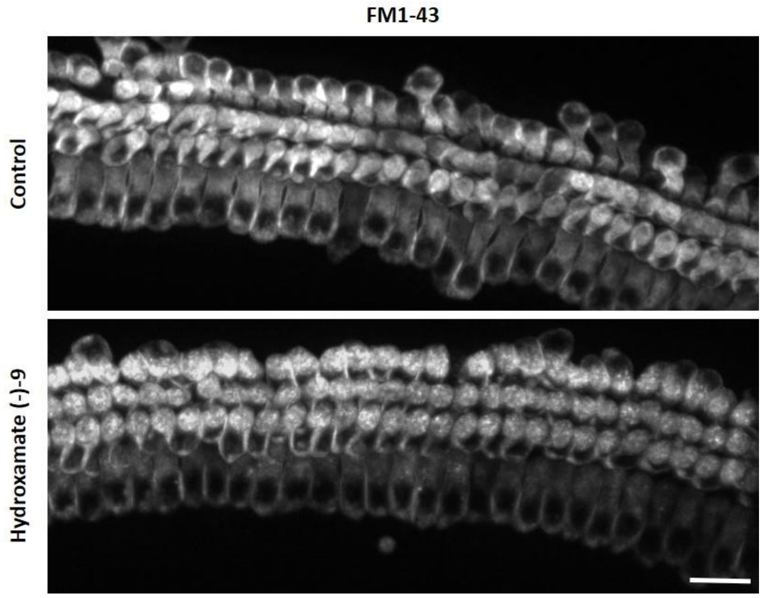


Supplementary figure 2 – **FM1-43 uptake is unchanged in the presence of hydroxamate (-)-9**. After treatment with 100nM hydroxamate (-)-9 for 14h, cochlear explants were exposed to 3µM of FM1-43 dye. FM1-43 labelling of the hair cells shows that hydroxamate (-)-9 does not block the mechanotransducer channels. Image correspond to middle of the basal end of the cochlear coil. Maximum intensity projection of a Z-stack spinning disc image is shown. Scale bar=10µm.
